# A retrospective review of colistin utilisation at a tertiary care academic hospital in South Africa

**DOI:** 10.4102/sajid.v36i1.205

**Published:** 2021-06-18

**Authors:** Liezl Majavie, Deanne Johnston, Angeliki Messina

**Affiliations:** 1 Department of Pharmacy and Pharmacology, Faculty of Health Sciences, University of the Witwatersrand, Johannesburg, South Africa; 2 Antimicrobial Stewardship unit, Netcare hospitals, Johannesburg, South Africa

**Keywords:** colistin, antimicrobials, antibiotics, colistin utilisation, multi-drug resistance, antibiotic resistance, antibiotic stewardship, human

## Abstract

**Background:**

The use of the antibiotic colistin was increasing as a treatment option for multidrug-resistant (MDR) infections. Standardisation of colistin dosing and more appropriate record-keeping practices were required to fully assess the optimal usage of colistin. The aim of this study was to determine how and why colistin was used in the treatment of MDR infections in a tertiary care public hospital in South Africa (SA).

**Methods:**

This cross-sectional retrospective record review described adult and paediatric patients who received colistin intravenously from 01 May 2016 to 31 April 2017. Information from patient records were captured on a data collection tool and analysed using descriptive statistics. Ethical approval was obtained from the Human Research Ethics Committee of the University of the Witwatersrand.

**Results:**

A total of 43 patient records were reviewed. *Acinetobacter baumannii* was the most common organism isolated (85.2% adults and 62.5% paediatrics). Colistin was mostly prescribed for sepsis (18 adults and 15 paediatrics). Most adults (66.7%) received loading doses as recommended; however, there was a great variation in maintenance doses. Paediatric patients reviewed also showed varying dosing according to weight. The mean duration of colistin therapy was 10 days. Carbapenems were most commonly co-administered with colistin (58%).

**Conclusion:**

The findings suggested that although colistin usage was restricted in the hospital, it was not adequately monitored or controlled. Doses prescribed were made at the discretion of prescribing doctors and differed to currently accepted guidelines. Improved record-keeping practices related to the monitoring of colistin use were required.

## Introduction

Colistin has widely been considered to be the last resort therapy for multidrug-resistant (MDR) infections.^1,2^ MDR infections are generally considered to be microbes, which display resistance to three or more antimicrobial classes.^3,4^ Microbial resistance to antibiotic classes such as cephalosporins, aminoglycosides, quinolones and carbapenems has contributed to an increase in the use of colistin globally.^5^ In South Africa (SA), colistin is used as salvage therapy for life-threatening infections,^1,2^ especially where resistance to the carbapenem class of antibiotics has been observed.^6^ The carbapenems have been considered to be part of the last line of defence against MDR gram-negative bacterial infections.^7^ Therefore, the increase in microbial resistance to carbapenems and other antibiotic classes has led to the increased use of colistin in SA.

Colistin, a polymyxin antibiotic, displays activity against MDR *Acinetobacter* and *Pseudomonas* as well as *Enterobacter aerogenes, Escherichia coli* (*E. coli*) and *Klebsiella* species.^6^ Colistin was discovered in the 1940s; however, reports of nephrotoxicity led to the decline in its use in the 1970s.^6,8^ High incidences of reported toxicities were as a result of inappropriate dosing regimens and are much more uncommon than previously thought.^9^ Adverse effects, such as nephrotoxicity and neurotoxicity, are largely considered to be dose dependent and reversible.^9^ Thus, dosing of colistin must be carefully monitored and its use preserved.^10^

The colistin formulation available in SA contains colistimethate sodium (CMS) equivalent to 150 mg colistin base activity (CBA) per vial. *In vivo* CMS, an inactive prodrug, is converted to the active colistin base.^6^

Different dosing units used across the world and variable pharmacokinetic profiles have resulted in much confusion in dosing regimens. The lack of harmonisation of dosing units has led to incorrect dosing of colistin and might have contributed to the development of resistance.^11^ However, recent evidence-based approaches to correct dosing of colistin have minimised the adverse effects and decreased the risk of resistance.^6,12^

Concern over resistance to colistin is reported both globally and in SA.^10,13^ To date, carbapenem-resistant Enterobacteriaceae (CRE) has demonstrated resistance to both colistin and tigecycline.^14^ It is proposed that few reports of colistin resistance in SA are because of a lack of surveillance rather than its low prevalence.^12^ The high use of colistin in poultry farming has led to the selection of the *mcr-1* gene found in *E. coli*.^15^ The first *mcr-1* gene recorded from *E. coli* strains was found in patients in Gauteng and Western Cape provinces in 2015.^13,14^

Colistin, an unregistered drug in SA, can be obtained on request from the South African Health Products Regulatory Authority (SAHPRA) as outlined in Section 21 of the *Medicines and Related Substances Control Act*.^1,6^ In the public health sector of SA, initial authorisation is granted by designated doctors at hospitals. Once completed, the forms are forwarded to SAHPRA for final approval.

South African-recommended adult dosing guidelines specify a loading dose (LD) of 9–12 MU (million units) regardless of kidney function and a maintenance dose of 3 MU every 8 h or 4.5 MU every 12 h, with adjustments made in renal impairment.^6^ Whilst the recommended dosing guidelines in children are based on weight, in general, 75 000–150 000 IU (international units) per kilogram per day in three divided doses is advised.^6^

Colistin usage is on the rise just as antibiotic resistance is increasing.^13^ For this reason, it is imperative to preserve its use for continuous efficacy. Scarce data are available regarding colistin utilisation in SA, particularly in the public sector and in the neonatal and paediatric patient population. Furthermore, a few studies have the opinion that limited data are available regarding its safety and efficacy profile.^9,16^ The lack of documented information on colistin use in Africa may in part be because of poor reporting and monitoring practices. This is a major concern as antibiotic resistance increases.^3^ Therefore, the aim of this research was to review the utilisation of colistin in a public sector, tertiary care hospital in SA and to determine why and how colistin is used in this setting.

## Methods

This study was a cross-sectional retrospective review of colistin utilisation at a public sector tertiary care hospital in Johannesburg, SA. It is one of the largest public sector hospitals with approximately 1068 beds.

The study reviewed records for a 01-year period (01 May 2016–31 April 2017) and included all patients who were prescribed colistin intravenously during the review period, including adult (> 18 years), paediatric (< 18 years) and neonatal (< 1 month) patients. Instances of incomplete data (missing of any information) were noted and recorded as findings.

Data were collected from Section 21 forms, patient files scanned onto the hospital database system and microbiological data from the National Health Laboratory Service (NHLS). The relevant information from these sources was entered onto a data collection sheet for each patient and captured on Microsoft Excel 2013 (Microsoft, USA).

Data recorded included patient demographics and characteristics, microbiological culture and susceptibility information as well as indications. The following was also recorded regarding colistin therapy: loading and maintenance doses, additional antibiotics administered (in addition to colistin), duration of therapy and overall patient outcome. Colistin doses and dosing frequencies prescribed were compared with the currently available local guidelines.^6,14^

### Statistical analysis

Data in this study were quantitatively analysed with descriptive statistics using STATA version 14.2 for Windows (StataCorp, 2015). All data gathered were summarised by indicating the distribution of data and measure of variability. Data normally distributed were expressed as mean ± standard deviation (SD), whilst those not normally distributed were described as median and frequency (*n*, %).

### Ethical considerations

Approval to conduct this study was obtained from the Gauteng Department of Health and hospital management. Ethical approval was granted by the University of Witwatersrand Human Research Ethics Committee (M170537).

## Results

A total of 62 Section 21 (order) forms were available and initially reviewed for the study period. Based on these forms, patient files were reviewed on the hospital scanning system. From the 62 Section 21 forms, 13 patients did not receive colistin although a Section 21 form was completed, and 6 patients’ files were not located. A total of 43 complete patient files were available for review and included in the study. A summary of demographic data of all patients who received colistin during the study period is presented in Table 1.

**TABLE 1:** Demographic data of patients in study (*n* = 43).

Variables	Patients	%
Adults	27	62.8
Paediatrics (< 18 years old)	16	37.2
**Age**		
Adults (years)	Median 39 years	-
Paediatric (days)	Median 22.5 days	-
**Gender**		
Female	20	46.5
Male	23	53.5
**Hospital ward**		
Intensive care unit	16	37.2
General medical wards	27	62.8

### Indications for colistin

Colistin was most frequently prescribed for sepsis, including 18 adult and 15 paediatric patients. Table 2 shows the diagnosis recorded for adult and paediatric patients included in this study.

**TABLE 2:** Diagnosis of patients studied as recorded in patient files.

Diagnosis	Paediatrics		Adults	
	*N* = 16	%	*N* = 27	%
Sepsis	15	93.8	18	66.7
Burns	1	6.3	-	-
Pleural effusion	-	-	3	11.1
Septic wound	-	-	3	11.1
Urinary tract infection	-	-	3	11.1

### Cultures

Colistin was prescribed in accordance with the organism isolated and corresponding antibiogram. The majority of specimen types were blood samples (65.1%).

Most frequently isolated organisms were *Acinetobacter baumannii* (85.2% in adults and 62.5% in paediatrics), *Pseudomonas aeruginosa* (3.7% in adults and 12.5% in paediatrics) and *Klebsiella pneumoniae* (18.8% paediatrics).

Review of the culture results supplied by the NHLS showed that multiple samples were frequently sent in for cultures for one patient within a 2-week period. These culture results often indicated that the same organism was cultured from different sites. Table 3 summarises the microbes found to be sensitive to antibiotics other than colistin.

**TABLE 3:** Additional antibiotics sensitive to isolated organisms in adults and paediatrics.

Additional antibiotics sensitive to isolated organism (possible alternative antibiotic to colistin) (*N* = 27)	Paediatric patients		Adult patients	
	Number	%	Number	%
Tigecycline	7	43.8	18	69.2
Aminoglycoside	6	37.5	5	19.2
Carbapenems	2	12.5	2	8.0
Cephalosporins	2	12.5	1	3.9
Fluoroquinolones	2	12.5	3	11.5
Piperacillin	2	12.5	1	3.9
Nitrofurantoin	-	-	1	3.9
Fosfomycin	-	-	1	3.9
Cotrimoxazole	3	18.8	-	-

### Compliance with colistin dosing recommendations

**FIGURE 1:**
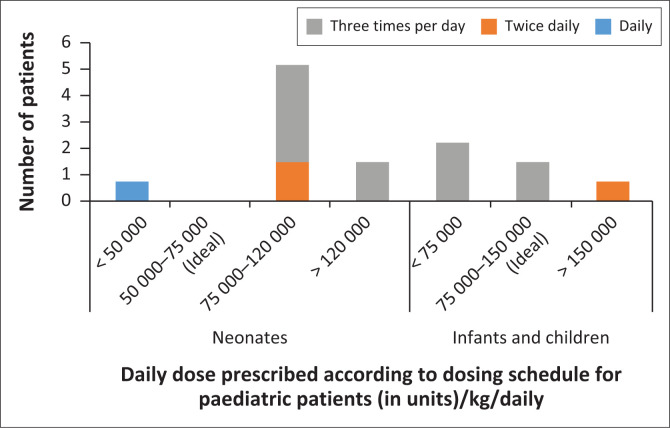
Doses of colistin administered in paediatric patients.

For the majority of paediatric patients, a sterile site culture (preferably blood or sterile aspirate) was obtained as recommended (93.8%) (Table 4). Of the paediatric patients studied, whose dosing is determined based on weight, nine patients (56.3%) were compliant to guidelines. Generally, paediatric maintenance dosages ranged between 75 000 units/kg/daily and 120 000 units/kg/daily, and the most common dosing interval was 8 hourly or 3 times a day (Figure 1). Current guidelines are not clear on how dose adjustments should be made in the case of renal impairment in paediatric patients. In the same group of patients, 3.69 doses were missed (SD 4.44, range 0–13) or not administered at dosing intervals as prescribed.

**TABLE 4:** Compliance with colistin dosing recommendations in paediatric and adult patients.

Recommendations	Number of paediatric patients (*N* = 16)	Number of adult patients (*N* = 27)
Obtain appropriate culture prior to the initiation of colistin therapy (blood cultures used)	15	13
Prescription of loading dose (LD)	1	22
Prescription of appropriate LD	Not recommended	18
**Prescription of appropriate maintenance dosing (MD)**		Because of the lack of eGFR data from the records reviewed, assessment on compliance to appropriate maintenance doses in adult patients could not be made
Neonates 50 000–120 000 IU/kg/day	7	
Infants and children 75 000–150 000 IU/kg/day	2	
Others (different to guideline†)	7	

† , Labuschagne Q, Schellack N, Gous A, et al. COLISTIN: Adult and paediatric guideline for South Africa, 2016. S Afr J Infect Dis. 2016;31(1):3–7. https://doi.org/10.4102/sajid.v31i1.95

LD, loading dose; eGFR, estimated glomerular filtration rate.

**FIGURE 2:**
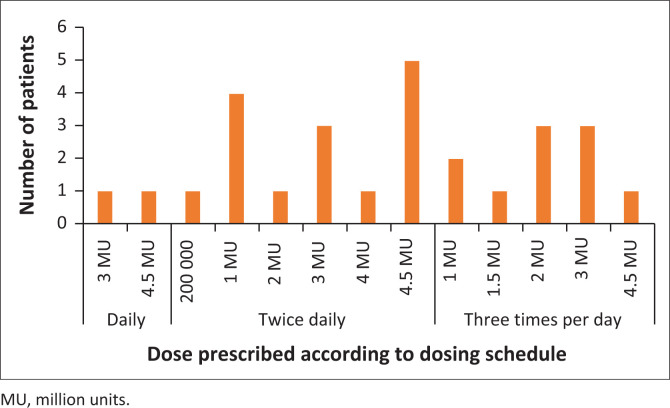
Doses and dosage frequency of colistin prescribed in adult patients (*n* = 27).

Most adults obtained appropriate LDs (81.5%) (Figure 2, Table 4). Maintenance doses varied considerably and were harder to interpret for appropriateness as renal function could not be determined from the data collection sources used in this study. In adults, 2.19 doses were missed (SD 2.77, range 0–13) or not administered at the prescribed dosing intervals for the course of colistin.

### Duration of colistin therapy

The mean duration of colistin therapy was 9.92 days (SD 4.68 days, range 1–16 days) for adult and 11.31 days for paediatric patients (SD 7.12 days, range 1–27 days).

### Co-administered antibiotics

In both adult and paediatric patients (Table 5), carbapenems were most commonly co-administered with colistin (58%, *n* = 25), followed by rifampicin 27.9% (*n* = 12). In adults, 63.0% (*n* = 17) also received co-trimoxazole. In 11 patients (25.6%), three antibiotics were concurrently administered (colistin plus two other agents).

**TABLE 5:** Co-administered antibiotics in paediatric and adult patients.

Antibiotic	Number of paediatric patients (*N* = 16)	Number of adult patients (*N* = 27)
Carbapenems (imipenem, ertapenem and meropenem)	9	16
Vancomycin	4	3
Macrolides (Azithromycin)	-	2
Aminoglycosides (Amikacin)	-	3
Tigecycline	-	2
Rifampicin	3	8
Co-trimoxazole	15	17

In most records reviewed, other antibiotics were given prior to the commencement of colistin, suggesting that one reason for the need to use colistin was because of treatment failure of antibiotics previously administered.

### Co-morbidities

Respiratory diseases and human immunodeficiency virus (HIV) were documented in 22.2% and 14.8% of adult patients, respectively. Fourteen patients (51.9%) had some degree of kidney injury or impairment.

Although not a co-morbidity, it was noted that 10 paediatric patients were born prematurely and six of these babies were noted to have very low birth weights (less than 1500 g).

### Length of stay and patient outcomes

The length of hospital stay for adult and paediatric patients was similar. The median stay of 47.5 days (IQR 30–83.5) was noted for adult patients and 47 days (IQR 37–70) for paediatric patients.

Overall, 48.8% of studied patients were discharged (14 adults and 7 paediatrics) and 41.9% of all patients demised (9 adults and 9 paediatrics). Of the adult patients, three (11.1%) were moved to another ward and one patient had an unknown outcome.

## Discussion

The findings suggest that although colistin usage is restricted, its use is not monitored or adequately controlled. Doses prescribed are made at the discretion of the prescribing doctor and varied considerably. This finding is similar to a colistin usage study in four private sector hospitals in SA, where it was noted that loading and maintenance doses were inconsistent and variable.^1^ To the best of our knowledge, this is the first time a study has exclusively focused on the review of colistin utilisation in a public sector hospital in SA, which includes both adult and paediatric patients. At a glance, it seems that colistin was infrequently prescribed and reserved for treatment in MDR infections. However, of concern is that not all patients’ records for which colistin was ordered could be located or were adequately completed.

One South African study indicated that colistin is used sparingly in the intensive care units (ICUs) of public hospitals.^17^ This was found to be the case in this study as only a total of 16 patients (37.2%) who received colistin were in ICU. However, this observation cannot be generalised to all SA public sector hospitals as the sample size was small and further studies are warranted to comment further on the extent of colistin consumption.

Although colistin was reserved for use in severely ill patients, where it was prescribed in accordance with culture results, the dosing of colistin did not always correlate with the local guidelines. Because of the retrospective nature of this study, it was not possible to ascertain if there were contributing reasons for doses prescribed, including the rationale for prescribing colistin when other antibiotics showed sensitivity.

It is well-accepted that an LD of colistin is recommended to ensure that optimal concentrations are quickly achieved to exert optimal bactericidal efficacy.^1^ It was encouraging to note that most adults (81.5%) reviewed in this study did receive LDs. This study did not have access to renal function tests to conclude if differing maintenance dosages were in line with the recommended dosages advised in renal impairment. However, the same LDs are recommended in all patients whether renally impaired or not. A total of 18 adult patients (66.7%) received appropriate LDs.^6^

Kift et al.^18^ stressed the need to avoid underdosing of colistin as this may lead to the development of resistance.

Colistin is a concentration-dependent bactericidal antibiotic; higher doses should be administered less frequently, because of its long half-life.^18^ The area under the plasma–concentration–time curve to the minimum inhibitory concentration (AUC:MIC) has been found to be a reliable parameter when calculating colistin efficacy.^6,19^ This study noted that doses were completely missed in certain instances or given later than the dosage intervals that were prescribed. A mean of 2.19 (SD 2.77, range 0–13) doses was missed for 27 courses of colistin given to adults. A mean of 3.69 (SD 4.44, range 0–13) doses was missed for 16 courses of colistin given to paediatric patients. This finding highlights the need for more robust education and monitoring of colistin to ensure that doses are administered when required, so that optimal and favourable therapeutic outcomes are achieved.

Combination therapy is currently advised as part of local guidelines.^6^ *In vitro* synergistic effects have been observed when colistin is used in combination with carbapenems.^20^ In this study, carbapenems were the most commonly co-administered antibiotics with colistin in 25 patients (16 adults and nine paediatrics).^6^ This finding is similar to a retrospective paediatric colistin usage study by Karaaslan et al.^21^ from 2011 to 2014 where it was found that colistin was usually co-administered with one other antibiotic. It was noted in the present study that co-trimoxazole was also frequently co-administered in adult (63%) and paediatric (93.8%) patients. This was most likely given as prophylactic therapy in immune compromised patients.

As expected, the majority of culture types were blood samples although other culture sites were also used. Culture results supplied by the NHLS showed that multiple samples were sent for cultures per patient. With regard to this, the NHLS advised that cultures sent in within a 2-week period were considered to be duplicate tests. In general, to obtain approval to use colistin, a blood culture result is required. This might have been the cause for repeat tests, as initial test was not performed on blood samples.

The most frequently isolated pathogens were *A. baumannii*, 76.7% in both adult and paediatric patients combined, followed by *P. aeruginosa* and *K. pneumoniae* at 7.0% each. These organisms are common nosocomial microbes.^5^ Similarly, a study on colistin usage in neonates in Turkey, by Cağan et al.,^9^ found that the most frequently isolated pathogens were *K. pneumoniae* and *A. baumannii* followed by *P. aeruginosa* and *E. cloacae*. The same study reported that patients treated with colistin were treated with at least one other antibiotic, a finding that is mirrored in this study.

In certain instances, colistin was prescribed although the sensitivity profile showed antibiotic sensitivity to another antibiotic. Tigecycline sensitivity was recorded in 69.2% of adult and 43.8% of paediatric patients.

Aminoglycoside sensitivity was recorded in 19.2% of adult and 37.5% of paediatric patients. This suggests that another antibiotic might have been used in accordance with the antibiogram (Table 3). In these cases, it was assumed that colistin was prescribed to optimise clinical outcome by using combination therapy with another agent, because patients’ severity of illness warranted the use of colistin or as a result of treatment failure of these other sensitive antibiotic agents.

### Study limitations and future recommendations

To align to more robust antibiotic stewardship strategies, the process of prescribing, dispensing and administering colistin should be revised and better recorded for adequate monitoring of its use in public sector hospitals. In accordance with recommendations made by Johnston et al.,^17^ an electronic system for dispensing medication should be implemented to adequately and timeously monitor antibiotic usage.^17^

Colistin usage was tracked from hardcopy Section 21 forms to scanned patient files. It is possible that some patients who received colistin during the study period were not recorded as their Section 21 form was not correctly filed in the pharmacy or a Section 21 form was lost or not completed at all. Also, some patient files were not located in the hospital scanning system or contained missing information. Future studies should consider prospective evaluation of patient records, which would also allow for possible interventions.

Although not a goal of this study, monitoring of side effects such as nephrotoxicity and neurotoxicity is necessary to monitor the safety profile of colistin. A major limitation of this study is that appropriateness of dose adjustments made in renal impairment was not assessed as estimated glomerular filtration rate (eGFR) values were not available within the records reviewed for all patients studied. Ooi et al. (2019) reiterated that renal function is a key determinant in optimal dosing in paediatric (and similarly adult) patients as the pharmacokinetics in paediatric patients revealed that clearances of colistin were related to creatinine clearance.^22^ Further research in this regard especially in paediatrics is pivotal.

## Conclusion

This study described the use of colistin in adult and paediatric patients admitted to a tertiary public hospital over a 1-year period. The use of colistin in this hospital was restricted for the critically ill where its use corresponded to culture and sensitivity results. The majority of patients received an LD and colistin was prescribed in combination with carbapenems. Although maintenance doses did not align to local guidelines, it could not be confirmed if these doses were adjusted because of renal function and as such, conclusions regarding appropriateness of therapy could not be made. The results from this study highlight areas for further investigations such as appropriate prescribing and dosing. Recommendations for practice include centralised record-keeping processes and appropriate dosing that may be facilitated through an interprofessional team approach consisting of at least prescribers, nurses, pharmacists and microbiologists.
